# Structural integrity of the anterior thalamic radiation predicts alpha oscillations and inattention during visual encoding

**DOI:** 10.1038/s41598-026-40086-5

**Published:** 2026-02-19

**Authors:** Joel P. Diaz-Fong, James McGough, James T. McCracken, Sandra K. Loo, Agatha Lenartowicz

**Affiliations:** 1 Semel Institute for Neuroscience & Human Behavior, Department of Psychiatry & Biobehavioral Science, University of California Los Angeles, 760 Westwood Plaza, Los Angeles, CA 90024 USA; 2 Institute of Medical Science, Temerty Faculty of Medicine, University of Toronto, Toronto, ON Canada; 3 Brain Health Imaging Centre, Centre for Addiction and Mental Health, Toronto, ON Canada; 4 Department of Psychiatry and Behavioral Science, University of California, San Francisco, San Francisco, CA USA

**Keywords:** ADHD, Inattention, Alpha oscillations, EEG, DTI, Neuroscience, Psychology, Psychology

## Abstract

Attention deficit/hyperactivity disorder (ADHD) is typically associated with working memory deficits, which are thought to arise from impaired attentional control. Previous research has highlighted abnormalities in alpha oscillations (8–12 Hz) during working memory tasks in children with ADHD, particularly attenuated event-related alpha power decreases (alpha ERD). However, the structural underpinnings of these oscillatory dynamics remain unclear. This study aimed to investigate the relationship between white matter microstructure and alpha modulation during a spatial working memory task in children with ADHD and typically developing (TD) controls. EEG and diffusion tensor imaging (DTI) data were analyzed from 115 children (ADHD *n* = 72; TD *n* = 43). We focused on three white matter tracts: the optic radiation (OR), anterior thalamic radiation (ATR), and the second branch of the superior longitudinal fasciculus (SLF2). DTI analyses revealed increased mean diffusivity in the ATR and SLF2 in ADHD, indicating reduced white matter integrity. Importantly, ATR microstructure significantly predicted alpha ERD, suggesting a key role for anterior thalamic pathways in modulating neural oscillations during working memory encoding. In contrast, SLF2 and OR microstructure did not significantly influence alpha modulation. These findings support a thalamus-mediated model of alpha modulation, where disruptions in anterior thalamic microstructural integrity contribute to attentional impairments in ADHD. Understanding these structural-functional relationships may inform targeted interventions aimed at improving executive function in this population.

**Supplementary Information:**

The online version contains supplementary material available at 10.1038/s41598-026-40086-5.

## Introduction

Working memory is a core neurocognitive function affected in attention deficit hyperactivity disorder (ADHD), impacting the ability to process, retain, and manipulate information over short periods^1^. These deficits are thought to arise from impaired attentional control, which has been linked to abnormal neural oscillatory activity, particularly in the alpha frequency band (8–12 Hz)^2^. Recent electroencephalography (EEG) studies have demonstrated that event-related decreases in alpha power (or alpha ERD) are reliably weaker (*Cohen’s d* = 0.44) in children with ADHD compared to typically developing (TD) controls^3–6^, highlighting its role as a putative marker of attentional impairment.

The functional role of alpha modulation in the visual cortex is hypothesized to include the selection or gating of sensory processing by selectively inhibiting or disinhibiting specific brain regions^7–10^. Additionally, concurrent EEG-fMRI studies, have confirmed that alpha ERD during visual encoding is associated with increased metabolic signal in occipital cortices^11,12^. Notably, in adolescents with ADHD, alpha ERD was more strongly associated with fronto-parieto-occipital connectivity^11^. This pattern has led to a proposed model of alpha ERD deficits in ADHD, which may indicate both ineffective activation of the visual cortex and aberrant fronto-parieto-occipital connectivity during visual encoding^2^.

While these studies highlight the neural underpinnings of working memory impairment in ADHD, it remains unclear whether the observed patterns reflect solely a functional phenomenon, such as imbalance in signaling at the synapse, a structural phenomenon, such as differences in white matter integrity, or both. Structural differences could influence axon propagation speeds, thereby affecting synchrony between distant cortical regions, ultimately constraining optimal cognitive performance^13,14^. Moreover, alpha ERD in the visual cortex may originate from multiple sources^15^, including reciprocal communication with the thalamus^16,17^ or long-range cortico-cortical connectivity carrying feedback information from higher-order association areas^18^. Thus, there exist multiple pathways, at both functional and anatomical levels, that could contribute to the visual encoding deficits during working memory in ADHD.

Diffusion tensor imaging (DTI) studies in ADHD have identified abnormalities in three key structural pathways particularly relevant to alpha modulation in the visual cortex: the optic radiation (OR)^19,20^, the anterior thalamic radiation (ATR)^21^, and the second branch of the superior longitudinal fasciculus (SLF2)^22^. These three pathways were selected because they represent distinct anatomical mechanisms by which alpha oscillatory activity in posterior cortex may be modulated during visual encoding. The OR, a component of the posterior thalamic radiation, connects the thalamus to the occipital cortex^23^, and maturation of this visual white matter pathway has been associated with age-related variation in alpha peak frequency^24^. The ATR consists of bidirectional projections from the anterior and medial thalamus, traveling through the anterior limb of the internal capsule to the prefrontal cortex^25,26^, positioning it to support higher-order attentional control and thalamic regulation of cortical excitability that may indirectly shape posterior alpha dynamics. The SLF2 connects the frontal part of the dorsal attention network to the parietal component of the ventral attention system and may serve as a direct prefrontal pathway for attentional processes^27^. Prior MEG studies have associated the SLF with alpha oscillations during visuospatial attention tasks^18,28 ^and one study has further suggested that SLF microstructure may mediate methylphenidate’s effect on beta oscillations in children with ADHD^29^. Together, these structures represent potential thalamocortical and cortico-cortical pathways underlying alpha oscillations during attentional and sensory processing.

Understanding how these candidate pathways contribute to alpha modulation in specific cognitive contexts, such as visual encoding, is critical for specifying the mechanisms contributing to attentional impairments in ADHD. In this study, we analyzed EEG and DTI data collected from children with ADHD and neurotypical peers to investigate whether microstructural properties in fronto-parieto-occipital regions are associated with alpha ERD during visual working memory encoding, and, if so, which of three candidate pathways (thalamic, thalamus-mediated, fronto-parietal cortical) accounts for the relationship. By identifying the neural circuits contributing to attentional control, this study sought to advance our understanding of the structural and functional mechanisms underlying working memory dysfunction in ADHD.

## Method

### Participants

Multimodal data was acquired from children enrolled in the Translational Research to Enhance Cognitive Control (TRECC) project^30–32 ^(ClinicalTrials.gov ID: NCT00429273). In total, 154 children (7–14 years) were identified as participating in both EEG and diffusion MRI procedures. ADHD participants were medication-naïve or not optimally treated with prior medication. All procedures were approved by the UCLA Institutional Review Board. Parents and participants provided written informed permission and assent prior to engaging in study procedures. The EEG data has been described in Lenartowicz et al.[3,4]. Full diagnostic details are provided in prior publications^30,32^.

### Behavioral and cognitive outcome measures

Data collection consisted of separate visits for the diagnostic interview, the MRI scan, and the EEG testing session. Neurodevelopmental disorder symptoms were assessed using the Kiddie Schedule for Affective Disorders and Schizophrenia-Present and Lifetime Version (KSADS-PL)^33^. Severity of ADHD symptoms was assessed with the Strengths and Weaknesses of ADHD Symptoms and Normal Behavior (SWAN) rating scale^34^. All children with ADHD had a Clinical Global Impression-Severity (CGI-S) score ≥ 4 and met full *DSM-IV* diagnostic criteria at the time of assessment.

### Spatial working memory task

**Fig. 1:**
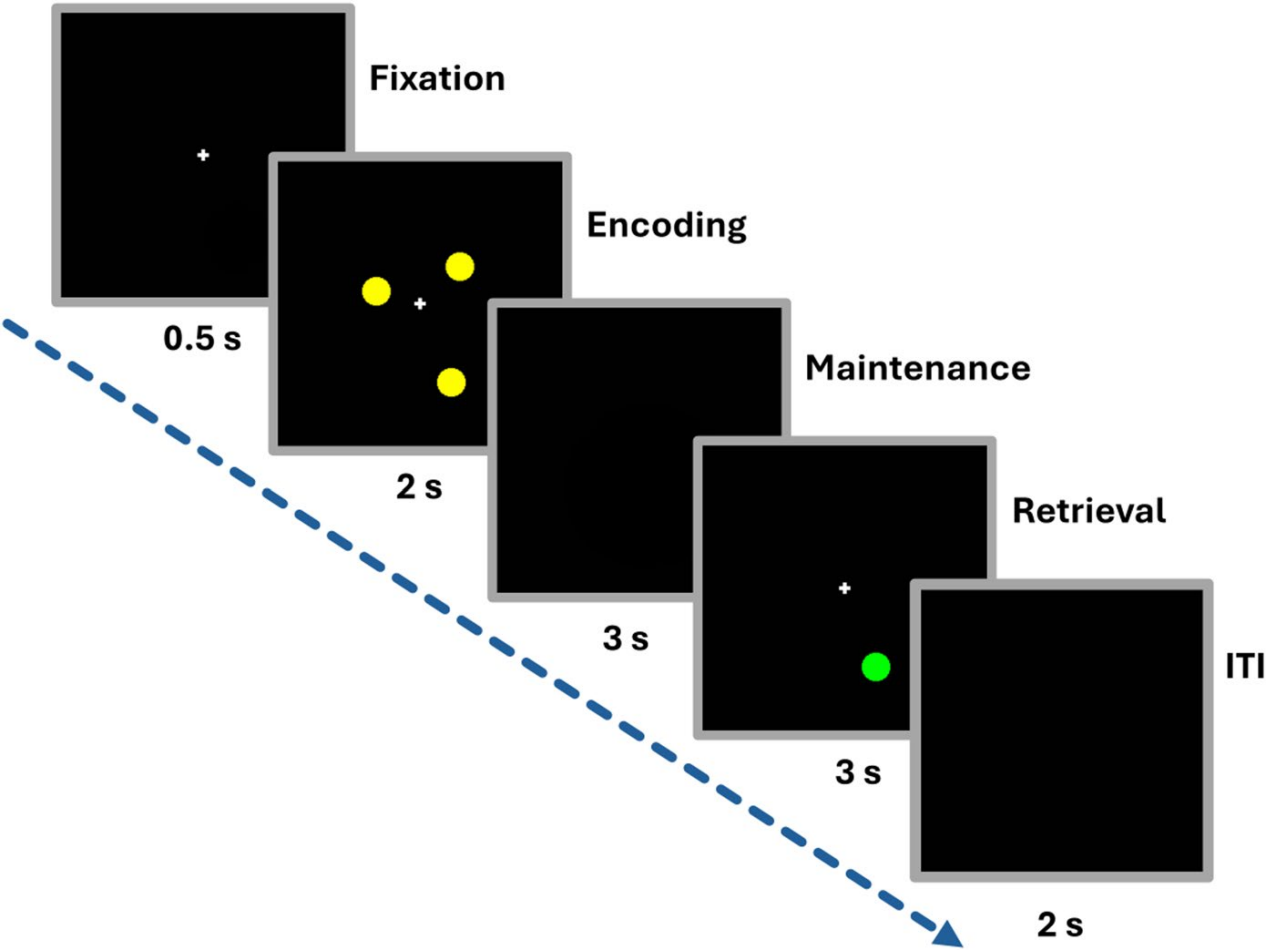
Spatial working memory delayed match-to-sample task. Participants encode the spatial location of 1, 3, 5 or 7 dots. After a maintenance interval, they indicate whether a green probe dot matches the location of any of the previously encoded yellow dots.

Participants were instructed to complete a computerized version of a spatial working memory delayed match-to-sample task^35,36 ^ (see Fig. 1). The task was administered using the E-Prime software (v1.1b5; Psychology Software Tools, Pittsburg, PA, United States) on a Dell PC (Round Rock, TX, United States). The task, designed to assess the ability to visually process and retain spatial information in working memory, consisted of three phases. During the *encoding* phase, participants were presented with a series of yellow dots (1, 3, 5, or 7 dots depending on the trial) randomly positioned on the computer screen and were instructed to memorize their locations. This was followed by a *maintenance* phase where, after the 2000 ms display, the screen went blank for 3000 ms, requiring participants to retain the spatial locations of the dots in memory. In the *retrieval* phase, a single green dot appeared, and participants had 3000 ms to respond whether the location of the green dot matched any of the previously shown yellow dots. This task setup allows for the examination of spatial working memory under varying cognitive loads, represented by the different numbers of dots.

### EEG acquisition & processing

EEG data were collected to assess neural activity while participants performed the spatial working memory task. A forty-channel Electro-cap (Electro-cap International, Inc., Eaton, OH) with silver-chloride electrodes was utilized. The electrodes were arranged in accordance with the international 10/20 System^37^. Data were recorded at 256 samples per second using the MANSCAN EEG system (SAM Technology, San Francisco, CA, United States). Electrode impedances were kept below ten kOhms and referenced to an average of single electrodes recorded separately at each ear lobe. To accurately model and analyze the scalp EEG data, individual 3-D electrode positions were digitized. This was achieved by measuring pairwise distances between the electrodes and key anatomical fiducials (the preauricular points and the nasion) using Fowler calipers.

EEG data processing was performed using an independent component analysis protocol (as described in Lenartowicz et al.[4]). Processing was performed using custom MATLAB (The MathWorks, Inc., Natick, MA, United States) scripts that utilized the EEGLAB toolbox (v.11.03.b)^38^. Briefly, the EEG data were high-pass filtered at 1 Hz, low-pass filtered at 54 Hz, noisy electrodes were removed by visual inspection, and the remaining channels were re-referenced to a common average reference. Following artefact correction, previously removed channels were interpolated using a spherical spline method to restore a common sensor montage across participants. For each participant’s data, trials were epoched time-locked to the onset of the encoding stimulus, and gross movements or artifacts were identified and removed if the signal power in an epoch exceeded the 85th percentile for more than 60% of the channels. Independent component analysis (ICA), using the extended *infomax* algorithm, was performed to decompose the data and remove artefacts from the signal. Alpha ERD during encoding was quantified by computing power in the alpha frequency range (8–12 Hz) from occipital electrodes (O1, O2, Oz) during stimulus encoding (0–2,000 ms). For each time point post stimulus-onset and within each frequency bin, power was normalized by dividing the value by the mean power during the baseline period preceding the fixation point for that trial (−600 to −100 ms). The resulting values were averaged across all occipital electrodes (O1, O2, Oz), frequencies in the alpha range (8–12 Hz), and time points within the encoding period (0 to 2000 ms) to produce a single value per subject.

### MRI acquisition

Neuroimaging MRI data were acquired on a Siemens 3 T *Allegra* scanner (Siemens, Erlangen, Germany) at the UCLA Brain Mapping Center. The diffusion MRI data were acquired as axial multislice single-shot echo-planar imaging (EPI) sequence (FOV 96; TR/TE = 7300/95 ms; flip angle = 90°; voxel size = 2.5 mm^3^; 5 directions at b = 0 *s/mm*^2^; 30 directions at b-value = 800 *s/mm*^2^)^39^. Anatomical scans were also acquired using a T1-weighted MPRAGE sequence (FOV 192; TR/TE = 2300/2.1 ms; voxel size = 1 mm^3^; 160 slices).

### Diffusion/Tractography

The multimodal analyses sought to test three alternative hypotheses regarding the relationship between white matter microstructure and alpha ERD: (i) white matter microstructure in the OR predicts alpha ERD supporting a thalamocortical model of alpha modulation through direct connections between thalamus and the visual cortex; (ii) ATR microstructure predicts alpha ERD, supporting a thalamus-mediated model, where feedforward control facilitates fronto-parietal interactions that, in turn, influence occipital activity; (iii) the SLF2 predicts alpha ERD, aligning with a direct prefrontal model of alpha modulation. Tract integrity for these three pathways was extracted as follows.

Fractional anisotropy (FA) and mean diffusivity (MD) were the primary measures of white matter microstructure. FA reflects the degree of directional coherence of water diffusion within white matter, where higher values indicate greater microstructural integrity, typically associated with well-myelinated and aligned axonal fibers. In contrast, lower FA suggests compromised white matter integrity, such as reduced axonal density or myelination. MD represents the overall magnitude of water diffusion within tissue, with higher MD values often indicating microstructural damage, such as axonal degeneration or increased extracellular space^40^.

The anatomical data were first preprocessed using FMRIB’s Software Library (FSL v6.0.4; www.fmrib.ox.ac.uk/fsl/)^41^. To leverage FSL’s advanced processing tools for diffusion data, an undistorted non-diffusion weighted image was synthesized from the T1-weighted image using Synb0-DisCo^42^. The susceptibility-induced off-resonance field was then estimated using FSL’s TOPUP (as described in Andersson et al.^43^). Eddy current-induced distortion correction^44^, outlier replacement^45^, and susceptibility-by-movement correction^46 ^was applied. Data quality assessment was performed by visual inspection of single-subject quality reports generated using FSL’s EDDY QUAD^47^. Bayesian estimation of diffusion parameters (BEDPOSTX) was used to model and determine the number of crossing fibres per voxel^48^. The diffusion tensor model was fitted using DTIFIT to derive FA and MD maps.

Anatomical and DTI images were analyzed in FreeSurfer (v7.4.1; https://surfer.nmr.mgh.harvard.edu/). Automated reconstruction of 42 white-matter pathways was performed using the TRACULA toolbox (Tracts Constrained by Underlying Anatomy)[49]. TRACULA employs global probabilistic tractography with anatomical neighborhood priors trained on high-quality annotated data and has been shown to perform robust reconstruction on lower resolution diffusion data^50^. Additionally, head motion measures were extracted, and a total motion index was calculated to use as a nuisance covariate in the analysis (as described in Yendiki et al.[51]; see Supplemental Materials for additional details). Average FA and MD measures were extracted from the three tracts of interest.

### Statistical analyses

All statistical analyses were performed in RStudio (R v4.1.1). Across analyses independent variables included DTI metrics (FA and MD) across alternate tracts (OR, ATR, SLF2), with the primary dependent variable being occipital alpha ERD during encoding. This allowed us to test if and which tracts predicts functional modulation in alpha oscillations during spatial working memory. The analyses were conducted in two phases to evaluate group effects on predictors, and functional relationships with alpha ERD as follows.

Group effects on DTI metrics (FA and MD) across tracts were examined to determine whether previous findings were replicable within our sample. To achieve this, separate MANCOVAs were conducted for each tract to reduce the number of comparisons and account for correlations between metrics. The multivariate outcomes included FA and MD within each of the three tracts. *Age*, *sex*, and *head motion* were included as covariates to control for potential confounding variables. For multivariate effects that reached significance, univariate analyses of covariance (ANCOVAs) were subsequently conducted for each dependent variable (e.g., FA or MD within a tract) to determine their specific contributions. These univariate tests were adjusted for the same covariates (age, sex, and head motion).

Next, to assess relationships between DTI metrics and alpha modulation during spatial working memory performance, linear models were used. This modeling framework allowed for the examination of both the main effects of DTI metrics and their interaction with *Group* on alpha modulation, while adjusting for *Age*. Thus, each model took the form: *Alpha ERD* ~ *DTI metric* + *Group* + *DTI metric* × *Group* + *Age*. To ensure interpretability of main and interaction effects, interaction terms were orthogonalized with respect to their associated main effects. This procedure preserves the variance attributed to the main effects and ensures that the interaction terms capture only unique moderation effects, reducing variance partitioning issues that can arise in models with correlated predictors^52,53^. Sex was not included as a covariate since prior analyses of EEG and DTI metrics revealed no significant effects of Sex. Additionally, FA and MD values were adjusted for head motion to account for its potential confounding influence. A significance threshold of *p* <.05 was used for all analyses. For tests involving DTI metrics (main effects), Benjamini-Hochberg false discovery rate (FDR) correction was applied to control for six comparisons (2 DTI metrics x 3 tracts).

**Fig. 2:**
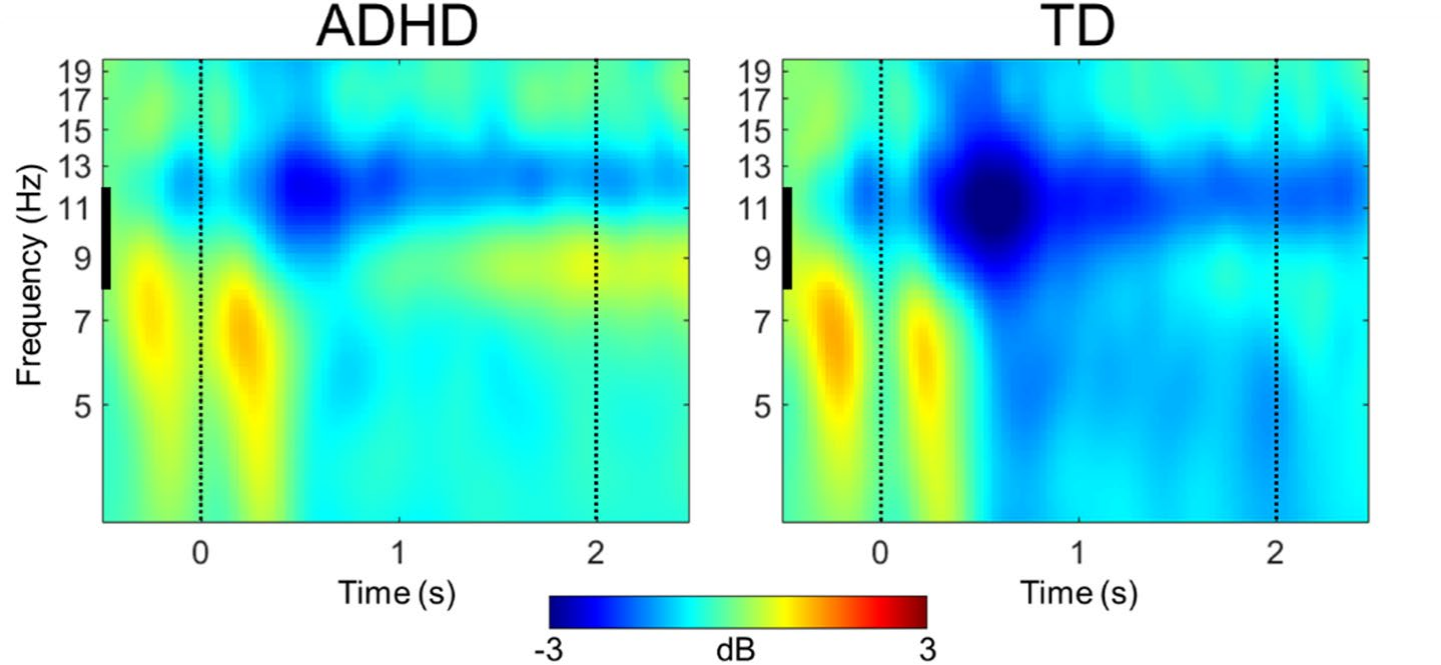
Alpha (8–12 Hz) power modulation in occipital electrodes (O1, O2, Oz) during stimulus encoding (0–2 s) by group. Power is expressed in decibels (dB) relative to a pre-stimulus baseline (− 600 to − 100 ms) for visualization; negative values reflect event-related decreases in alpha power. ADHD = attention deficit/hyperactivity disorder; TD = typically developing controls.

Finally, because this study includes a subsample of participants previously reported in earlier EEG studies^3,4^, we conducted a replication analysis of alpha ERD during the spatial working memory task. For this analysis, we opted to work in channel space rather than ICA source space and applied a task accuracy cutoff of ≤ 50% (instead of 60%) to retain more participants in the sample. Additionally, we averaged across all memory loads (1, 3, 5, and 7 dots) to simplify the model. Full details of the analysis and results are provided in the Supplemental Materials and in Fig. 2.

## Result

### Participant characteristics

Of the 154 children initially identified, 115 with both EEG and DTI data were included in the final analysis after exclusions for incomplete scans, excessive motion during MRI acquisition, poor EEG quality, or task accuracy ≤ 50% (see Supplemental Materials for more details). The larger sample with usable DTI (*N* = 133) was used for group comparisons of DTI metrics (Table 1).

**Table 1:** Demographics for the full sample (*N* = 133).

Variable	ADHD (*n* = 86)	TD (*n* = 47)	Group comparison
Sex (male/female), *n*	59/27	29/18	*χ*^2^(1) = 0.647, *p* =.421
Age (years)	10.05 ± 1.99	10.77 ± 2.25	*t*(85) = 1.84, *p* =.069
Full Scale IQ	102.73 ± 12.52	107.55 ± 14.49	*t*(84) = 1.92, *p* =.058
SWAN			
Inattention	14.92 ± 6.72	35.98 ± 8.46	*W* = 3968, *p* <.001
Hyperactivity	22.14 ± 10.08	36.68 ± 9.42	*W* = 3460, *p* <.001

Note. Values are mean ± standard deviation unless otherwise indicated. ADHD = attention deficit/hyperactivity disorder; TD = typically developing controls; SWAN = Strengths and Weaknesses of ADHD Symptoms and Normal Behavior scale (higher scores indicate fewer symptoms); χ² = chi-square test; *t* = Welch two-sample *t*-test; *W* = Wilcoxon rank-sum test. Non-parametric tests were used for variables that violated normality assumptions.

Demographic characteristics, including sex distribution and age, did not significantly differ between groups (see Table 2). However, the TD group had significantly higher Full Scale IQ scores compared to the ADHD group (*t*(82.65) = 2.11, *p* =.038). In terms of task performance, ADHD participants showed significantly lower task accuracy compared to TD participants (70.7% vs. 79.8%, *W* = 2229, *p* <.001). There were no significant differences in mean response time between groups (*W* = 1458.5, *p* =.607), but ADHD participants displayed significantly greater response time variability compared to TD participants (470 ms vs. 401 ms, *t*(97.57) = −3.60, *p* <.001).

**Table 2:** Demographics and task performance for the combined EEG-DTI sample (*N* = 115).

Variable	ADHD (*n* = 72)	TD (*n* = 43)	Group comparison
Sex (male/female), *n*	47/25	27/16	*χ*^2^(1) = 0.073, *p* =.788
Age (years)	10.15 ± 1.97	10.91 ± 2.29	*W* = 1871, *p* =.062
Full Scale IQ	102.96 ± 12.67	108.40 ± 13.78	*t*(82.65) = 2.11, *p* =.038
SWAN			
Inattention	15.07 ± 6.85	36.58 ± 8.56	*W* = 3035, *p* <.001
Hyperactivity	22.68 ± 9.78	37.58 ± 9.13	*t*(93.33) = 8.24, *p* <.001
Task performance			
Accuracy (%)	70.65 ± 11.48	79.8 ± 10.73	*W* = 2229, *p* <.001
Mean response time (ms)	1324	1303	*W* = 1458.50, *p* =.607
Response time variability (ms)	470	401	*t*(97.57) = −3.60, *p* <.001

Note. Values are mean ± standard deviation unless otherwise indicated. Mean response time reflects the per-subject average reaction time, and response time variability refers to the within-subject standard deviation of reaction time. ADHD = attention deficit/hyperactivity disorder; TD = typically developing controls; SWAN = Strengths and Weaknesses of ADHD Symptoms and Normal Behavior scale (higher scores indicate fewer symptoms); χ² = chi-square test; *t* = Welch two-sample *t*-test; *W* = Wilcoxon rank-sum test. Non-parametric tests were used for variables that violated normality assumptions.

### Replication analysis of alpha ERD

Analysis of alpha power during stimulus encoding revealed a significant main effect of Group (*F*(1,111) = 4.228, *p* =.042), with ADHD participants exhibiting weakened alpha modulation compared to TD participants (see Fig. 2). Additional details of the results are provided in the Supplemental Materials.

### Group differences in white matter microstructure

**Fig. 3:**
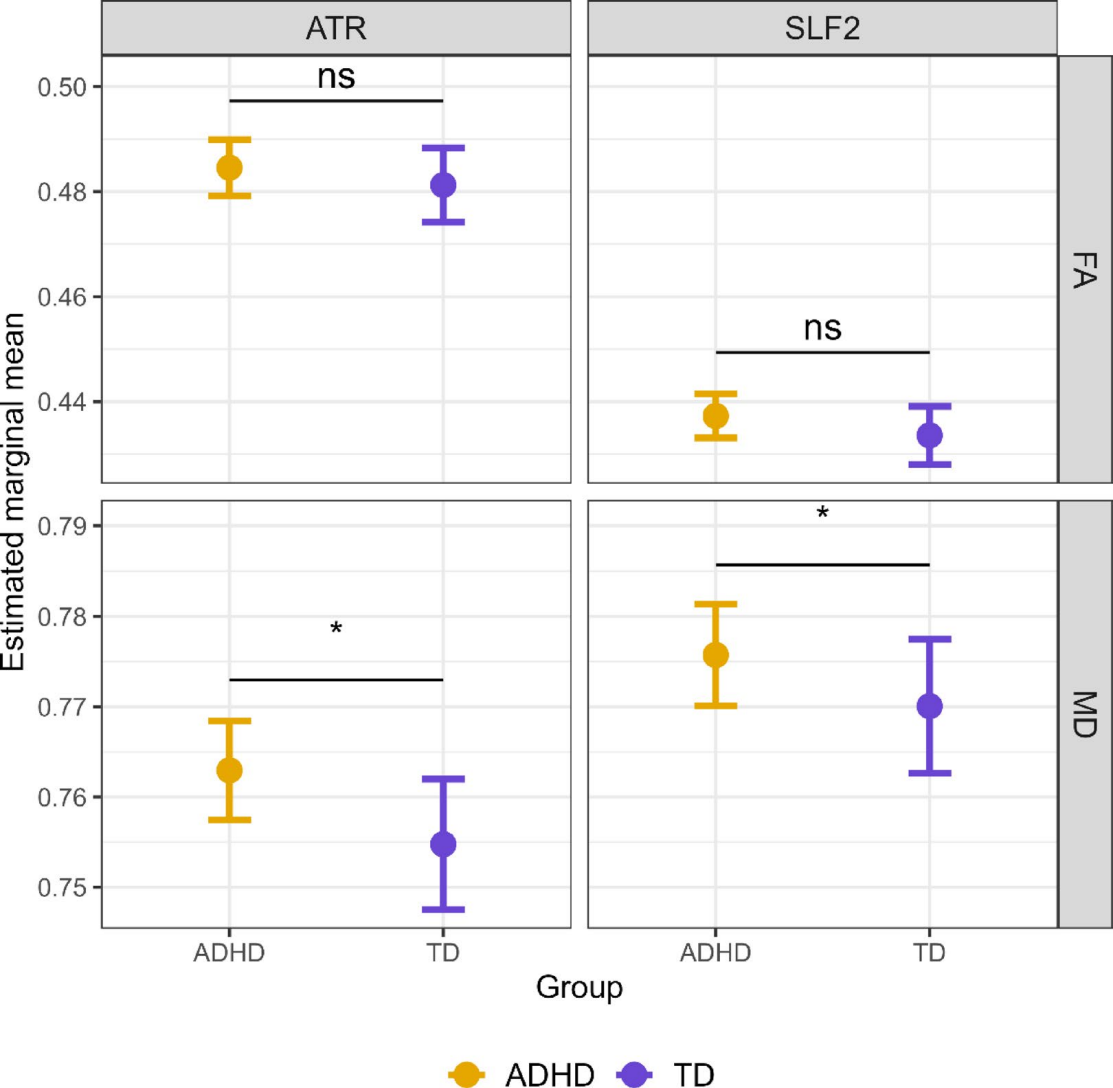
Estimated marginal means of fractional anisotropy (FA; unitless) and mean diffusivity (MD; ×10⁻³ mm²/s) in the anterior thalamic radiation (ATR) and superior longitudinal fasciculus II (SLF2) by group (TD vs. ADHD). Values represent model-derived marginal means adjusted for age, sex, and head motion. MD values are scaled by ×10^3^ for visual comparability. Error bars represent 95% confidence intervals derived from ANCOVA models. Significance markers indicate group differences based on univariate follow-up tests from a prior MANCOVA (ns = not significant; * = *p* <.05). ADHD = attention deficit/hyperactivity disorder; TD = typically developing controls.

Multivariate analyses for white matter microstructure revealed significant group differences. For the SLF2, significant multivariate effects were observed for group (V = 0.065, *F*(2,127) = 4.39, *p* =.014) and age (V = 0.218, *F*(2,127) = 17.70, *p* <.001), while head motion effects were nonsignificant (V = 0.023, *F*(2,127) = 1.50, *p* =.23). Univariate analysis showed that group differences were significant for MD_SLF2_ (*F*(1,128) = 5.16, *p* =.025) but not in FA_SLF2_ (*F*(1,128) = 0.004, *p* =.95). The MD_SLF2_ was larger for kids with ADHD than peers without ADHD (see Fig. 3). This pattern of findings was also present in the ATR. Significant multivariate effects were observed for group (V = 0.0627, *F*(2,127) = 4.25, *p* =.016) and age (V = 0.155, *F*(2,127) = 11.64, *p* <.001), with motion approaching significance (V = 0.046, *F*(2,127) = 3.07, *p* =.05). Subsequent univariate analyses indicated significant group differences for MD_ATR_ (*F*(1,128) = 6.83, *p* =.01) but not FA_ATR_ (*F*(1,128) = 0.22, *p* =.64), with MD_ATR_ larger for kids with ADHD than for peers without ADHD. Note that, motion significantly influenced FA_ATR_ (*F*(1,128) = 4.65, *p* =.033), though it had no effect on MD_ATR_ (*F*(1,128) = 0.07, *p* =.80). In contrast to the SLF2 and ATR, no significant multivariate group effects were observed for the OR (V = 0.016, *F*(2,127) = 1.02, *p* =.36), though age (V = 0.11, *F*(2,127) = 7.91, *p* <.001) and motion (V = 0.103, *F*(2,127) = 7.25, *p* =.001) had significant effects. Across all MANCOVA and ANCOVA analyses, Sex was not significant and was therefore not included as a covariate in subsequent models. Thus, the SLF2 and ATR, but not the OR, showed significant group differences in MD, indicating higher diffusivity in children with ADHD compared to their peers, which may reflect compromised white matter integrity.

### Relationship between white matter microstructure and alpha modulation

**Fig. 4:**
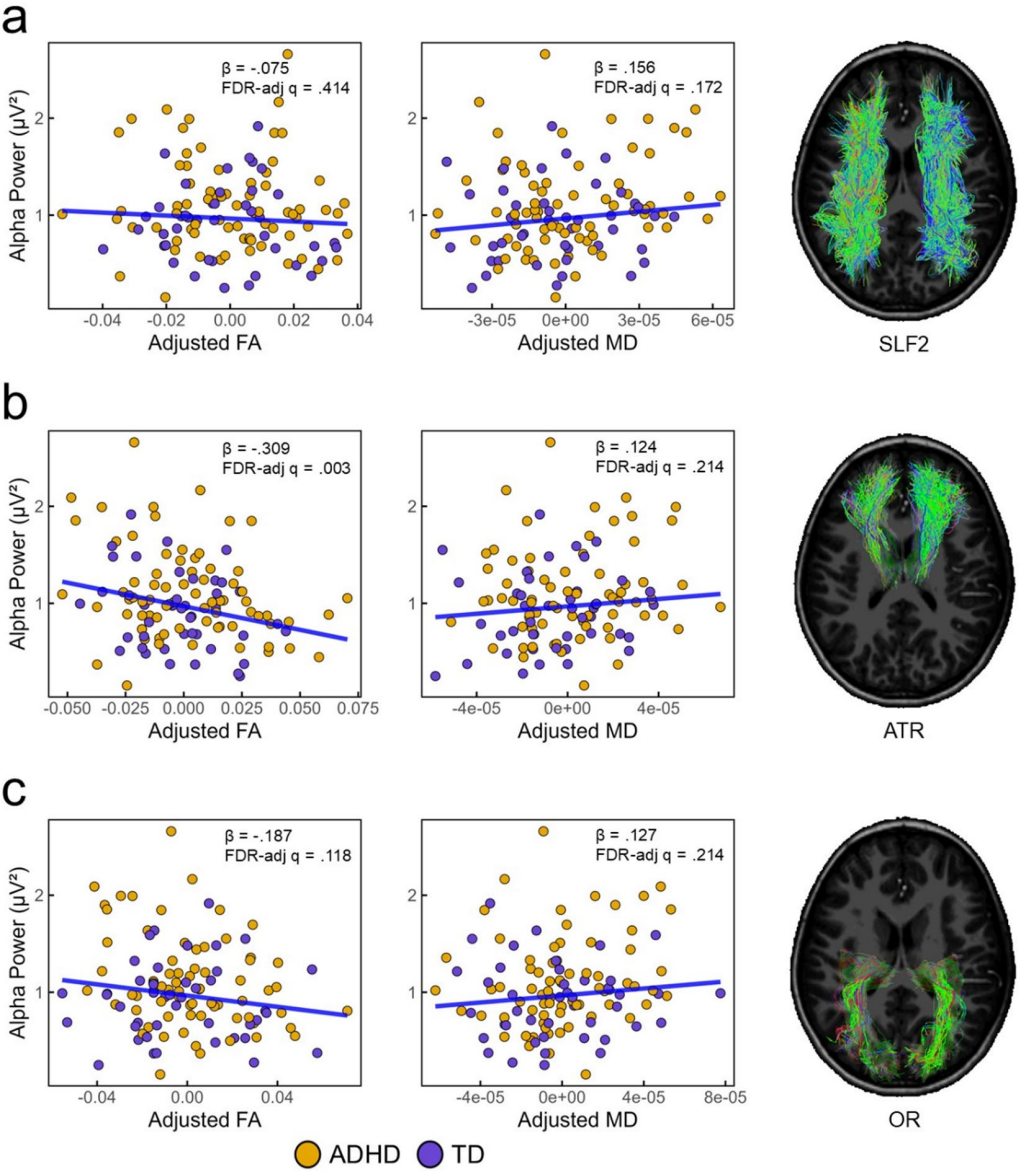
Associations between occipital alpha power and white matter microstructure across tracts. Alpha power is expressed in microvolts squared (µV²) and baseline-corrected. Fractional anisotropy (FA) and mean diffusivity (MD) values reflect residualized estimates from linear models controlling for age and head motion. Tract reconstructions (right) from a representative participant illustrate the corresponding white matter pathways. (**a**) Relationship between occipital alpha power and FA (left column) and MD (right column) in the superior longitudinal fasciculus II (SLF2). (**b**) Corresponding associations for the anterior thalamic radiation (ATR). (**c**) Corresponding associations for the optic radiation (OR). ADHD = attention deficit/hyperactivity disorder; TD = typically developing controls.

Linear models were used to assess whether DTI metrics accounted for variance in alpha power modulation (see Fig. 4). For the SLF2, neither FA (β = − 0.075, *t*(110) = − 0.820, *p* =.414, FDR_adjusted_ *q* = 0.414) nor MD (β = 0.156, *t*(110) = 1.724, *p* =.087, FDR_adjusted_ *q* = 0.172) were significantly associated with alpha power modulation. In contrast, FA_ATR_ showed a significant negative association with alpha power (β = − 0.309, *t*(110) = −3.568, *p* =.001, FDR_adjusted_ *q* = 0.003), such that higher FA_ATR_ was associated with stronger alpha power modulation (see Fig. 4b). The association between MD_ATR_ and alpha power did not reach significance (β = 0.124, *t*(110) = 1.355, *p* =.178, FDR_adjusted_ *q* = 0.214). For the OR, FA showed a nominally significant negative association at the uncorrected level (β = − 0.187, *t*(110) = −2.084, *p* =.039), but this did not survive FDR correction (FDR_adjusted_ *q* = 0.118). MD_OR_ was not significantly associated with alpha modulation (β = 0.127, *t*(110) = 1.407, *p* =.162, FDR_adjusted_ *q* = 0.214). No significant interactions between DTI metrics and group were observed for any of the three tracts. Taken together, these results indicate that microstructural properties of the ATR, specifically FA, contribute to individual differences in alpha modulation during task performance, whereas associations in other tracts did not survive correction for multiple comparisons.

### Associations between anterior thalamic radiation, inattention, and task performance

Given prior associations between alpha modulation with inattention symptoms and task performance measures^3,4^, we first conducted correlation analyses to explore whether FA_ATR_ showed direct associations with these measures. Spearman rank correlations revealed no significant associations between FA_ATR_ and SWAN Inattention (*ρ* = − 0.02, *p* =.82) or task accuracy (*ρ* = 0.04, *p* =.66). Likewise, Pearson’s correlation indicated no significant relationship between FA_ATR_ and response time variability (*r* = −.10, *p* =.30).

**Fig. 5:**
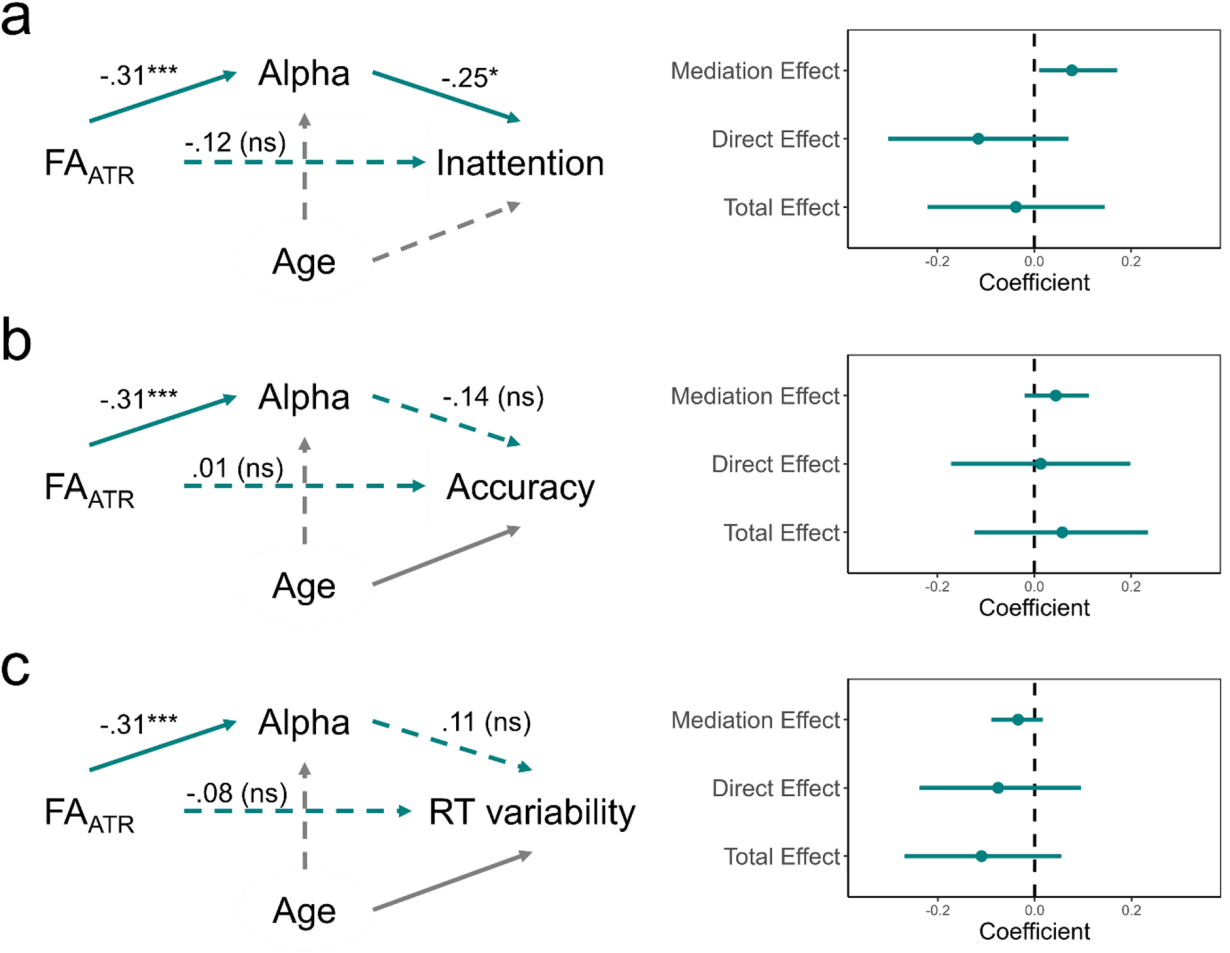
Schematic representation of the path models testing the effects of fractional anisotropy of the anterior thalamic radiation (FA_ATR_) on behavioral performance and symptom measures through occipital alpha power. Left panels: solid arrows denote significant paths and dashed arrows denote nonsignificant paths. Right panels: plots display standardized coefficients (β) and bias-corrected bootstrap confidence intervals for the mediation, direct, and total effects, controlling for age. (**a**) Inattention: greater FA_ATR_ predicted stronger alpha modulation, which in turn was associated with fewer inattentive symptoms, yielding a significant indirect effect. (**b**) Accuracy: the path from alpha power to task accuracy was nonsignificant, and no indirect effect of FA_ATR_ was supported. (**c**) Response time (RT) variability: the path from alpha power to response time variability was nonsignificant, and no indirect effect of FA_ATR_ was supported. FA_ATR_ values were adjusted for head motion prior to modeling. Significance levels: ns = not significant; * = *p* <.05; *** = *p* <.001.

Given the absence of direct associations, we next tested whether alpha modulation mediated the relationship between FA_ATR_ and inattentive symptom severity. Path analyses were conducted in R using the *lavaan* package^54^, which estimates indirect, direct, and total effects within a structural equation modeling framework. In a model including age as a covariate, FA_ATR_ (adjusted for motion) significantly predicted alpha modulation (β = − 0.31, *p* =.001), and alpha modulation was positively associated with inattentive symptoms (β = − 0.25, *p* =.022). Because higher SWAN scores reflect fewer inattentive symptoms, this finding indicates that greater alpha modulation was linked to lower inattentive symptom severity. The indirect effect of FA_ATR_ on inattentive symptoms via alpha was positive and supported by bias-corrected bootstrap confidence intervals based on 10,000 resamples (β = 0.078, 95% CI [0.010, 0.171]), whereas the direct and total effects of FA_ATR_ on symptoms were nonsignificant. These results indicate that FA_ATR_ relates to inattentive symptoms indirectly through its effect on alpha modulation (Fig. 5).

We also tested accuracy and reaction time variability as outcomes in analogous path models. Neither analysis revealed a significant indirect effect of FA_ATR_ on task performance via alpha modulation (Accuracy: β = 0.044, 95% CI [−0.020, 0.113]; Response time variability: β = − 0.034, 95% CI [−0.090, 0.017]), and total effects were non-significant. Thus, the mediation pathway appeared specific to inattention symptoms rather than task-level performance measures.

## Discussion

In the present study, we investigated the relationship between white matter microstructure and alpha modulation during spatial working memory encoding in children with and without ADHD. Specifically, we focused on three white matter tracts—the OR, ATR, and the SLF2—given their established roles in connecting fronto-parieto-occipital regions involved in attention and sensory processing. Our results demonstrate several important findings. First, our DTI analysis revealed significant group differences in the ATR and SLF2, but not in the OR. Specifically, children with ADHD showed elevated MD in the ATR and SLF2 compared to TD peers, indicating reduced white matter integrity in these tracts, but not the direct connection between thalamus and posterior cortex. Importantly, microstructural properties of the ATR, but not the SLF2 or OR, were significantly associated with alpha ERD during task performance across all participants. These findings suggest that the ATR may play a key role in modulating neural oscillations related to attention and working memory as well as playing a putative role in corresponding deficits in ADHD.

Our findings of increased MD in the ATR and SLF2 among children with ADHD are consistent with a prior DTI study^21^. High MD values may reflect microstructural alterations such as disorganized white matter or myelin breakdown^40^. Interestingly, we observed no significant group differences in FA, possibly due to our inclusion of head motion as a nuisance regressor, which may mitigate spurious group differences arising from movement artifacts^51^. In our sample, head motion significantly influenced FA in the ATR and the OR. This is in line with previous research^29, ^which also found that head motion was predictive of higher FA values in the ATR, albeit only in the control group. Such motion-related effects on FA may explain the inconsistent group findings in the literature^55^.

The observed relationship between ATR microstructure and alpha modulation supports a thalamus-mediated model of attentional control effects on alpha power. The ATR’s bidirectional connections between the thalamus and prefrontal cortex may facilitate both feedforward and feedback processes that regulate occipital alpha oscillations during task performance. These findings align with prior studies implicating the thalamus in both sensory processing and attentional control^56^. Moreover, greater ATR FA predicted stronger alpha modulation, which in turn was associated with fewer inattentive symptoms. Path analyses confirmed that the effect of FA_ATR_ on inattentive symptoms was indirect, operating through alpha rather than directly from FA_ATR_ to symptoms, and appeared specific to inattentive symptom severity rather than task-level performance measures. This aligns with the idea that structural connectivity constrains electrophysiological dynamics, which in turn shape cognition^13,14^. Thus, disruptions in anterior thalamic microstructural integrity may hinder the transmission of feedforward signals necessary for the thalamus to effectively regulate cortical oscillations and synchrony patterns critical for sustained cognitive engagement^57–59^. Collectively, these findings provide novel evidence that microstructural variations in anterior thalamic pathways predict neural oscillatory dynamics during visual working memory encoding, which in turn relate to inattentive symptom severity, underscoring their potential role in the pathophysiology of ADHD.

More broadly, this thalamus-mediated constraint on task-related alpha modulation may be relevant to alpha-band abnormalities reported in other neurodevelopmental and sensory atypical populations. In autism spectrum disorder, reduced posterior alpha power and attenuated alpha ERD have been reported, with evidence that baseline alpha levels predicts the magnitude of task-related modulation^60^. Consistent with recent frameworks emphasizing alpha oscillations as a flexible, task-dependent control signal rather than a fixed deficit, these findings suggest that large-scale thalamocortical control pathways may constrain the range over which posterior alpha can be dynamically modulated during goal-directed processing^15^. In contrast, reduced posterior alpha activity observed following sight recovery after congenital blindness has been interpreted as reflecting disrupted development of alpha-generating circuitry itself^61^, highlighting that alpha abnormalities may arise either from altered circuit formation or from limitations in the control mechanisms that regulate otherwise intact posterior networks. The present findings are more consistent with the latter account.

By comparison, the OR, which connects the thalamus to the occipital cortex^23^, showed an apparent relationship with alpha ERD; however, this effect did not survive correction for multiple comparisons. Given the OR’s primary role in visual information transfer, this trend may nonetheless be noteworthy. Recent evidence suggests that resting-state alpha peak frequency is related to the maturation of this visual white matter pathway^24^, raising the possibility that the OR contributes to the development of oscillatory dynamics in posterior regions. However, our findings do not provide strong evidence for a role of the OR in task-related alpha modulation, at least within the present sample.

While we found increased MD in the SLF2 in children with ADHD compared to TD controls, this tract’s microstructural properties were not significantly associated with alpha ERD. This result is somewhat unexpected, given prior studies that have found associations between SLF and neural oscillations during visuospatial attention tasks^18,28,29^. Although the SLF2 is a critical component of the dorsal attention network^27^, the absence of significant DTI–EEG associations in our study may indicate that this tract is more closely related to oscillations during cued attentional orienting than to the neural oscillatory dynamics underlying visual working memory encoding. Furthermore, most previous DTI studies in ADHD have not differentiated between the three branches of the SLF. In this study, we specifically examined SLF2, and our findings of increased MD in children with ADHD suggest that SLF2 integrity is compromised. This distinction is important, as inconsistent definitions of the SLF across studies may contribute to variable findings and confusion regarding its structure and function^62^. For example, previous versions of FreeSurfer used the terms “temporal SLF” and “posterior SLF” to define the arcuate fasciculus and the third branch of the SLF, respectively^49^, adding complexity to the interpretation of SLF findings in ADHD. Future studies should explore whether different branches of the SLF contribute differentially to attentional mechanisms in ADHD, particularly under varying tasks and cognitive loads.

Several limitations should be noted. First, the exclusion of participants due to motion artifacts and poor EEG data quality may have introduced a selection bias, potentially affecting the generalizability of our findings. However, it is also possible that this bias is informative, as such exclusions may disproportionately remove more hyperactive individuals, effectively enriching the sample for participants with predominantly inattentive symptom profiles^3^. Future studies should explicitly test this possibility by comparing across ADHD subtypes or symptom profiles (e.g., inattentive vs. hyperactive/impulsive) and comorbidity patterns, rather than relying solely on larger undifferentiated samples. Replication in larger cohorts, including adolescents and adults with ADHD, will also be important for establishing the generalizability of these structural-functional relationships across development. Importantly, the cross-sectional design limits causal inferences about the relationship between white matter microstructure and neural oscillations. Longitudinal studies could provide more definitive insights into how these neural pathways develop over time in individuals with ADHD and whether interventions can modify these relationships. Another limitation is that we analyzed signals at the sensor level from occipital electrodes rather than performing source modeling, to avoid excluding participants who would have been removed based on our prior ICA clustering procedure (see Lenartowicz et al.[3,4]). Consequently, the measured signals are likely to reflect a mixture of neural sources, not exclusively occipital activity. Finally, the diffusion data were collected using a single-shell sequence with 30 directions, which limits the ability to model crossing fibers or more complex microstructural properties. To address these limitations, future studies should incorporate high-density EEG recordings and multi-shell acquisition of diffusion data which would allow for more advanced modeling of the functional and structural dynamics between EEG and diffusion metrics.

Overall, our study provides novel insights into the structural underpinnings of alpha modulation during working memory encoding in children with and without ADHD. We replicated significant group differences in white matter microstructure and extended prior work by demonstrating for the first time that the anterior thalamic radiation plays a critical role in modulating alpha oscillations during visual working memory encoding. These findings highlight the importance of thalamocortical microstructural integrity in the attentional impairments that are characteristic of ADHD. Our findings of associations between white matter microstructure and neural oscillatory dynamics advance the understanding of the structural-functional mechanisms underlying attentional impairments in ADHD.

## Supplementary Information

Below is the link to the electronic supplementary material.

Supplementary Material 1

## Data Availability

The data that support the findings of this study are available on request from the corresponding author. The data is not publicly available due to privacy or ethical restrictions.
