# Supplementary material for: Structural integrity of the anterior thalamic radiation predicts alpha oscillations and inattention during visual encoding
Source: Sci Rep. 2026 Feb 19;16:9905. doi: 10.1038/s41598-026-40086-5 (PMC13018530; doi:10.1038/s41598-026-40086-5)
Supplement: Supplementary file 1 — Supplementary Material 1 [file 41598_2026_40086_MOESM1_ESM.pdf]

Supplementary Material

Title
Structural integrity of the anterior thalamic radiation predicts alpha oscillations and inattention during visual encoding

Authors
Joel P. Diaz-Fong[1-3], James McGough[1], James T. McCracken[1,4], Sandra K. Loo[1‡], Agatha Lenartowicz[1‡]

[1]Semel Institute for Neuroscience & Human Behavior, Department of Psychiatry & Biobehavioral Science, University of California Los Angeles, Los Angeles, California, United States
[2]Institute of Medical Science, Temerty Faculty of Medicine, University of Toronto, Toronto, Ontario, Canada
[3]Brain Health Imaging Centre, Centre for Addiction and Mental Health, Toronto, Ontario, Canada
[4]Department of Psychiatry and Behavioral Science, University of California, San Francisco, San Francisco, California, United States

[‡]These authors contributed equally as senior authors.

Supplementary Methods

Replication of Previous EEG Results with Current Subsample

Total Motion Index (TMI)

Supplementary Tables

Table S1. Multivariate analysis of covariance (MANCOVA) results for group differences in DTI metrics.

Table S2. Post hoc ANCOVA results for group differences in fractional anisotropy (FA) and mean diffusivity (MD) for tracts showing significant effects in the MANCOVA.

Table S3. Linear model results for alpha modulation predicted by superior longitudinal fasciculus-2 (SLF2) fractional anisotropy (FA), group, and age.

Table S4. Linear mixed model results for alpha modulation predicted by superior longitudinal fasciculus-2 (SLF2) mean diffusivity (MD), group, and age.

Table S5. Linear mixed model results for alpha modulation predicted by anterior thalamic radiation (ATR) fractional anisotropy (FA), group, and age.

Table S6. Linear mixed model results for alpha modulation predicted by anterior thalamic radiation (ATR) mean diffusivity (MD), group, and age.

Table S7. Linear mixed model results for alpha modulation predicted by optic radiation (OR) fractional anisotropy (FA), group, and age.

Table S8. Linear mixed model results for alpha modulation predicted by optic radiation (OR) mean diffusivity (MD), group, and age.

Supplementary Figures

Figure S1. Flowchart of data exclusion for final statistical analysis

Figure S2. Superior longitudinal fasciculus-2 (SLF2) tract reconstruction in a single participant, generated using TRACULA.

Figure S3. Anterior thalamic radiation (ATR) tract reconstruction in a single participant, generated using TRACULA.

Figure S4. Optic radiation (OR) tract reconstruction in a single participant, generated using TRACULA.

Figure S5. Histograms of the four motion metrics and the total motion index

Supplementary References

Supplementary Methods

Replication of Previous EEG Results with Current Subsample

To examine the effects of working memory load and group on alpha modulation during stimulus encoding, a two-way analysis of covariance (ANCOVA) was conducted. The model included Group, with covariates for Sex and Age. Analysis of alpha power during stimulus encoding revealed a significant main effect of Group ($F(1,111) = 4.228$, $p = .042$), with ADHD participants exhibiting weakened alpha modulation compared to TD participants. Age significantly influenced alpha power ($F(1,111) = 5.245$, $p = .024$), but no significant effect was observed for Sex ($F(1,111) = .346$, $p = .558$).

Total Motion Index (TMI)

The total motion index (TMI) is a composite metric that quantifies motion artifacts by standardizing and aggregating multiple motion-related metrics such as average translation, average rotation, percent bad slices, and average dropout scores (Yendiki et al., 2014). Each metric is normalized using a z-scoring method based on the interquartile range (IQR). The TMI is then computed as the sum of all valid z-scores:

$$TMI_i = \sum_{i=1}^{4} \frac{x_{ij} - M_j}{IQR}$$

Where j indexes the four motion metrics
x_i is the observed value of the motion metric j
M is the median of j metric
IQR is the interquartile range (i.e., $Q_3 - Q_1$, the difference between the 75th and 25th percentiles).

Supplementary Tables

Table S1. Multivariate analysis of covariance **(**MANCOVA) results for group differences in DTI metrics.

	**Effect/Term**	**Pillai**	**F-value**	**df1**	**df2**	**p-value**
**SLF2**	Group	.064683	4.392	2	127	.014 *
	Covariate (Age)	.217990	17.701	2	127	< .001 ***
	Covariate (Sex)	.044583	2.963	2	127	.055 .
	Covariate (Motion)	.023078	1.500	2	127	.227
**ATR**	Group	.062708	4.248	2	127	.016 *
	Covariate (Age)	.154962	11.645	2	127	< .001 ***
	Covariate (Sex)	.027845	1.819	2	127	.166
	Covariate (Motion)	.046175	3.074	2	127	.050 *
**OR**	Group	.015859	1.023	2	127	.362
	Covariate (Age)	.110745	7.908	2	127	< .001 ***
	Covariate (Sex)	.018726	1.212	2	127	.301
	Covariate (Motion)	.102513	7.253	2	127	.001 **

Note. SLF2 = superior longitudinal fasciculus-2; ATR = anterior thalamic radiation; OR = optic radiation.

Table S2. Post hoc ANCOVA results for group differences in fractional anisotropy (FA) and mean diffusivity (MD) for tracts showing significant effects in the MANCOVA.

	**Effect/Term**	**F-value**	**df1**	**df2**	**p-value**
**FA_{SLF2}**	Group	.004	1	128	.953
	Covariate (Age)	30.967	1	128	< .001 ***
	Covariate (Sex)	.000	1	128	.998
	Covariate (Motion)	1.913	1	128	.169
**MD_{SLF2}**	Group	5.163	1	128	.025 *
	Covariate (Age)	28.154	1	128	< .001 ***
	Covariate (Sex)	3.359	1	128	.069 .
	Covariate (Motion)	.015	1	128	.903
**FA_{ATR}**	Group	.220	1	128	.640
	Covariate (Age)	20.908	1	128	< .001 ***
	Covariate (Sex)	3.504	1	128	.064 .
	Covariate (Motion)	4.650	1	128	.033 *
**MD_{ATR}**	Group	6.830	1	128	.010 *
	Covariate (Age)	15.851	1	128	< .001 ***
	Covariate (Sex)	.597	1	128	.441
	Covariate (Motion)	.067	1	128	.796

Note. FA = fractional anisotropy; MD = mean diffusivity; SLF2 = superior longitudinal fasciculus-2; ATR = anterior thalamic radiation; OR = optic radiation.

Table S3. Linear model results for alpha modulation predicted by superior longitudinal fasciculus-2 (SLF2) fractional anisotropy (FA), group, and age.

Effect	β	SE	df	t-value	p-value	95% CI	FDR_{adj} q
Intercept	.89	.06	110	15.57	< .001	[.78, 1.00]	-
FA	-.03	.03	110	-.82	.414	[-.10, .04]	.414
Group	.12	.07	110	1.66	.100	[-.02, .26]	-
FA*Group	.01	.07	110	.07	.943	[-.14, .15]	-
Age	-.08	.04	110	-2.27	.025 *	[-.15, -.01]	-

Note. β = unstandardized regression coefficient; SE = standard error; CI = confidence interval; FA = fractional anisotropy; SLF2 = superior longitudinal fasciculus-2; FDR_{adj} = Benjamini–Hochberg false discovery rate corrected p-value (reported for FA only). p-values for other predictors are uncorrected.

Table S4. Linear model results for alpha modulation predicted by superior longitudinal fasciculus-2 (SLF2) mean diffusivity (MD), group, and age.

Effect	β	SE	df	t-value	p-value	95% CI	FDR_{adj} q
Intercept	.90	.06	110	15.85	< .001	[.79, 1.01]	-
MD	.06	.03	110	1.72	.087	[-.01, .13]	.175
Group	.11	.07	110	1.48	.142	[-.04, .25]	-
MD*Group	.04	.07	110	.60	.553	[-.10, .19]	-
Age	-.08	.03	110	-2.37	.020 *	[-.15, -.01]	-

Note. β = unstandardized regression coefficient; SE = standard error; CI = confidence interval; MD = mean diffusivity; SLF2 = superior longitudinal fasciculus-2; FDR_{adj} = Benjamini–Hochberg false discovery rate corrected p-value (reported for MD only). p-values for other predictors are uncorrected.

Table S5. Linear model results for alpha modulation predicted by anterior thalamic radiation (ATR) fractional anisotropy (FA), group, and age.

Effect	β	SE	df	t-value	p-value	95% CI	FDR_{adj} q
Intercept	.88	.05	110	16.23	< .001	[.77, .99]	-
FA	-.12	.03	110	-3.57	.001 **	[-.18, -.05]	.003 **
Group	.14	.07	110	1.97	.051	[.00, .27]	-
FA*Group	.05	.08	110	.70	.488	[-.10, .20]	-
Age	-.08	.03	110	-2.47	.015 *	[-.15, -.02]	-

Note. β = unstandardized regression coefficient; SE = standard error; CI = confidence interval; FA = fractional anisotropy; ATR = anterior thalamic radiation; FDR_{adj} = Benjamini–Hochberg false discovery rate corrected p-value (reported for FA only). p-values for other predictors are uncorrected.

Table S6. Linear model results for alpha modulation predicted by anterior thalamic radiation (ATR) mean diffusivity (MD), group, and age.

Effect	β	SE	df	t-value	p-value	95% CI	FDR_{adj} q
Intercept	.90	.06	110	15.77	< .001	[.79, 1.02]	-
MD	.05	.03	110	1.36	.178	[-.02, .12]	.214

Group	.10	.07	110	1.34	.185	[-.05, .24]	-
MD*Group	-.04	.07	110	-.60	.552	[-.19, .10]	-
Age	-.08	.02	110	-2.38	.019 *	[-.15, -.01]	-

Note. β = unstandardized regression coefficient; SE = standard error; CI = confidence interval; FA = fractional anisotropy; MD = mean diffusivity; FDR_{adj} = Benjamini–Hochberg false discovery rate corrected p-value (reported for MD only). p-values for other predictors are uncorrected.

Table S7. Linear model results for alpha modulation predicted by optic radiation (OR) fractional anisotropy (FA), group, and age.

Effect	β	SE	df	t-value	p-value	95% CI	FDR_{adj} q
Intercept	.89	.06	110	15.80	< .001	[.77, 1.00]	-
FA	-.07	.03	110	-2.08	.039 *	[-.14, .00]	.118
Group	.13	.07	110	1.79	.077	[-.01, .27]	-
FA*Group	-.07	.07	110	-1.03	.307	[-.21, .07]	-
Age	-.08	.03	110	-2.23	.028 *	[-.15, -.01]	-

Note. β = unstandardized regression coefficient; SE = standard error; CI = confidence interval; FA = fractional anisotropy; OR = optic radiation; FDR_{adj} = Benjamini–Hochberg false discovery rate corrected p-value (reported for FA only). p-values for other predictors are uncorrected.

Table S8. Linear model results for alpha modulation predicted by optic radiation (OR) mean diffusivity (MD), group, and age.

Effect	β	SE	df	t-value	p-value	95% CI	FDR_{adj} q
Intercept	.89	.06	110	15.83	< .001	[.78, 1.01]	-
MD	.05	.03	110	1.41	.162	[-.02, .12]	.214
Group	.11	.07	110	1.58	.117	[-.03, .26]	-
MD*Group	.07	.07	110	1.08	.283	[-.06, .21]	-
Age	-.08	.03	110	-2.40	.018 *	[-.15, -.01]	-

Note. β = unstandardized regression coefficient; SE = standard error; CI = confidence interval; MD = mean diffusivity; OR = optic radiation; FDR_{adj} = Benjamini–Hochberg false discovery rate corrected p-value (reported MD only). p-values for other predictors are uncorrected.

Supplementary Figures

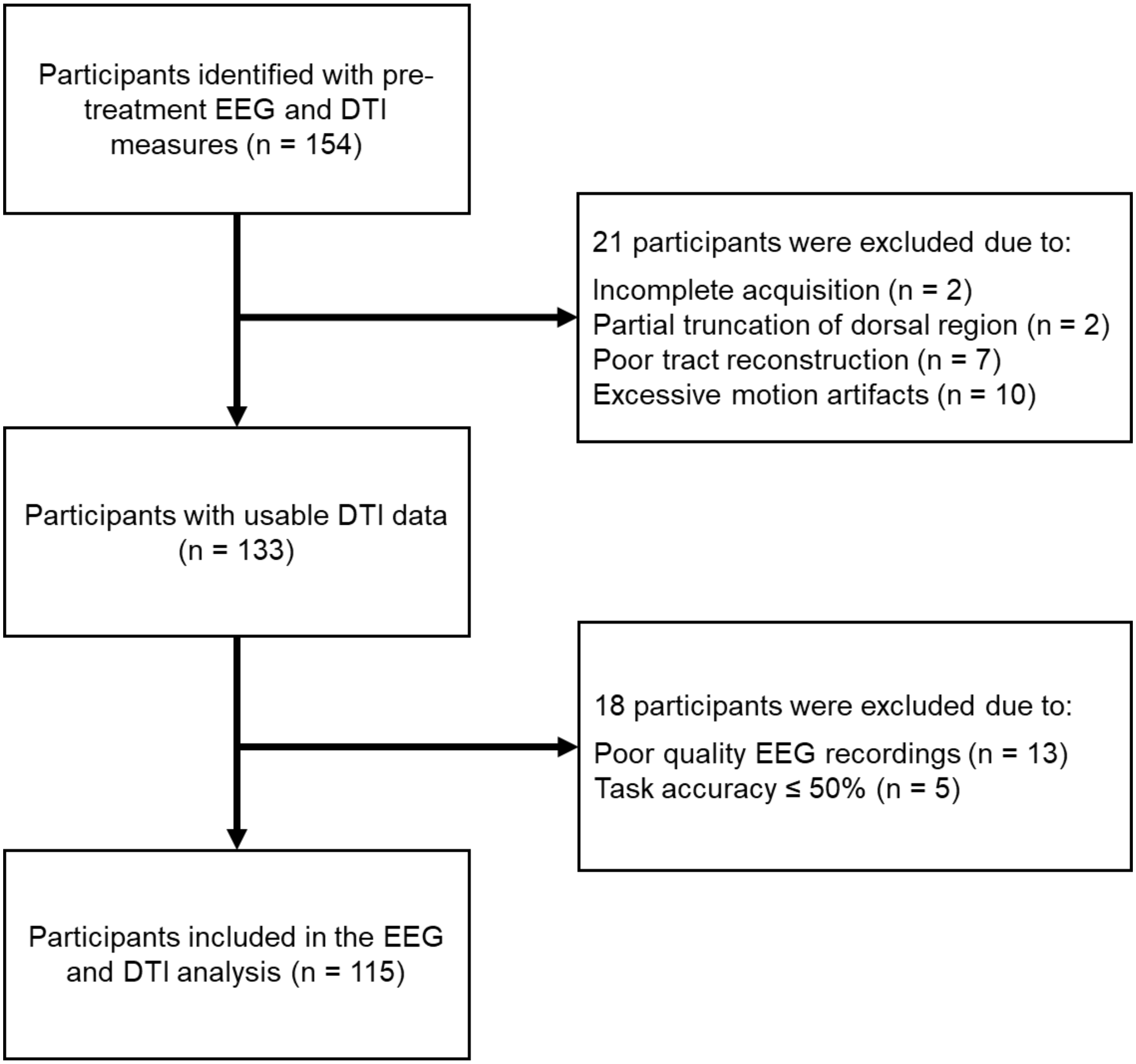

Figure S1. Flowchart of data exclusion for final statistical analysis. Out of the 154 children initially identified with pre-treatment baseline measures, 21 were excluded due to excess motion or incomplete scans. Of the remaining 133 children, 13 were excluded due to poor EEG recordings and 5 were excluded because task accuracy was ≤ 50%. In total, 115 children with both EEG and DTI were included in the analysis. The larger sample with usable DTI (N=133) were considered for group comparisons on DTI metrics.

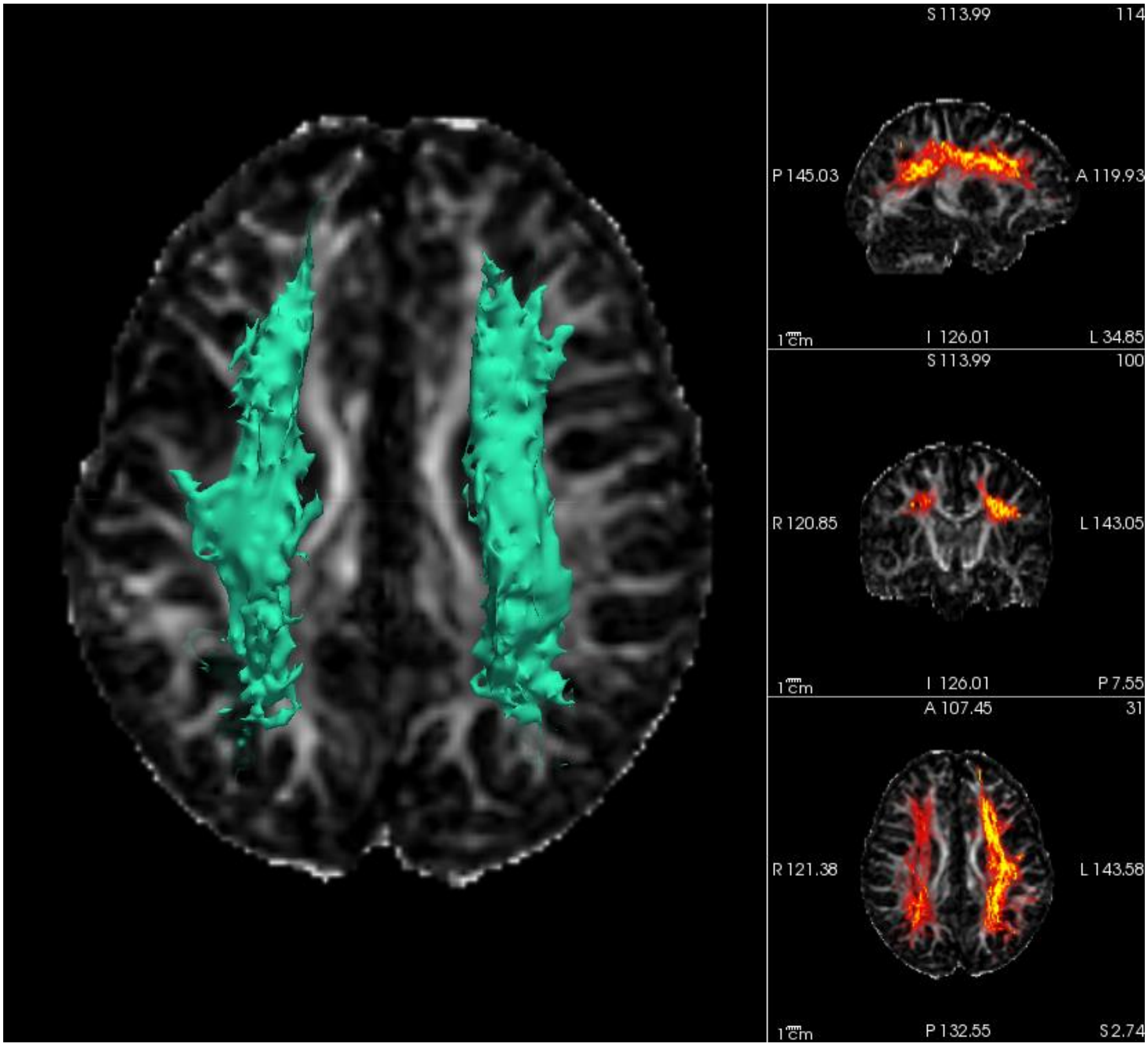

Figure S2. Superior longitudinal fasciculus II (SLF2) tract reconstruction in a single participant, generated using TRACULA.

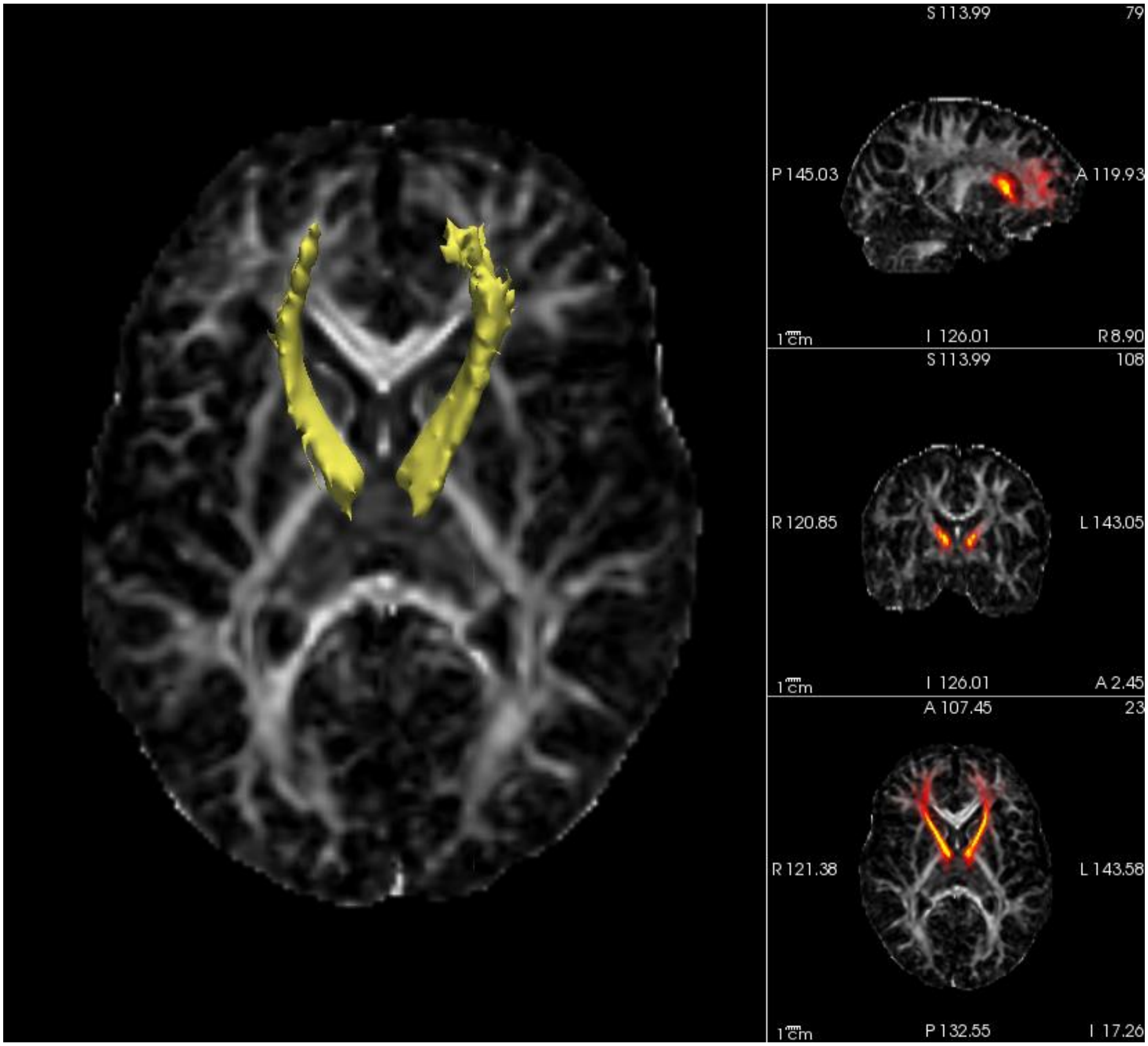

Figure S3. Anterior thalamic radiation (ATR) tract reconstruction in a single participant, generated using TRACULA.

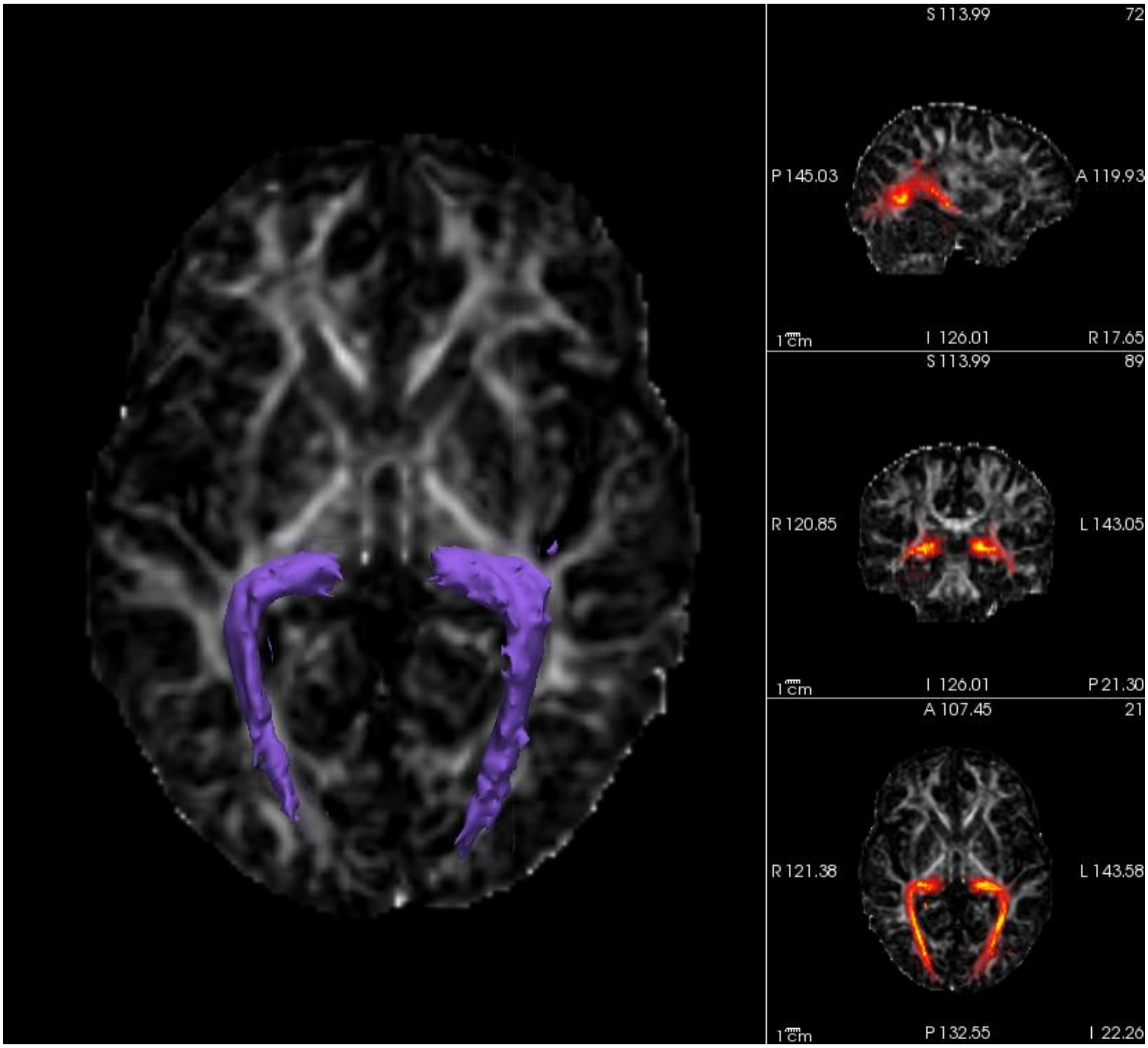

Figure S4. Optic radiation (OR) tract reconstruction in a single participant, generated using TRACULA.

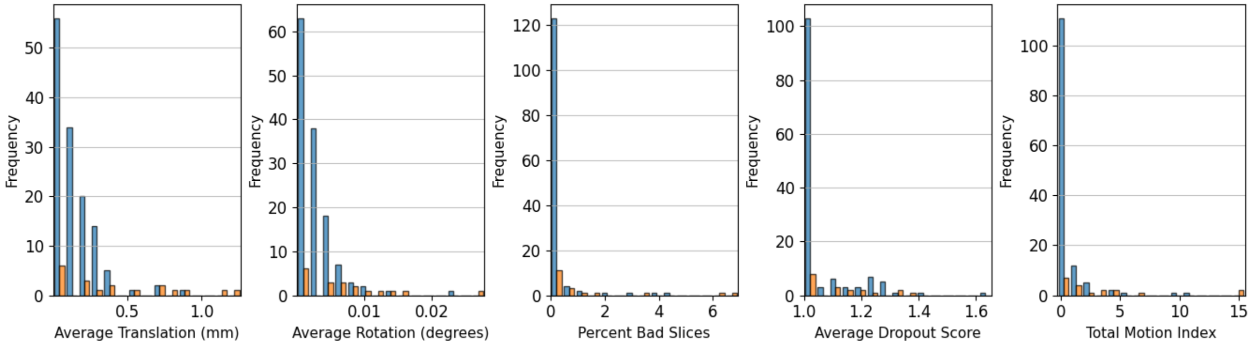

Figure S5. Histograms of the four motion metrics and the total motion index for the 133 participants included in our analysis (blue) and the 19 participants excluded due to poor tract

reconstruction or excessive motion (orange). Each histogram displays the distribution of a motion metric, with separate counts for included and excluded participants.

Supplementary References

Yendiki, A., Koldewyn, K., Kakunoori, S., Kanwisher, N., & Fischl, B. (2014). Spurious group differences due to head motion in a diffusion MRI study. *NeuroImage*, *88*, 79–90. https://doi.org/10.1016/j.neuroimage.2013.11.027
